# Consistent performance of cytomegalovirus IgG assays across platforms contrasts with marked IgM variability, supporting IgG avidity–based infection classification in a high-prevalence population

**DOI:** 10.1038/s41598-026-50663-3

**Published:** 2026-04-29

**Authors:** Farah Trad, Marah Abdallah, Nouran Zein, Salma Younes, Parveen B. Nizamuddin, Nadin Younes, Hisham ElBanawy, Baheieh Al-Abbadi, Mansour Al-Hiary, Zain Abou-Nouar, Oraib Al-Subeihi, Yaser Al-Zubi, Ahmad Al-Manaseer, Anwar Al-Jaloudi, Ahmed Ismail, Wanida Laiwattanapaisal, Pattramon Aungbamnet, Pollanat Loungjinda, Palanee Ammaranond, Hadi M. Yassine, Houssein Ayoub, Laith J. AbuRaddad, Gheyath K. Nasrallah

**Affiliations:** 1 Department of Biomedical Science, College of Health Sciences, QU Health, Qatar University, P.O. Box 2713, Doha, Qatar; 2 Biomedical Research Center, QU Health, Qatar University, P.O. Box 2713, Doha, Qatar; 3 Al Borg Diagnostics, PO Box 5207, Doha, Qatar; 4 Department of Immunopathology Princess Iman Bint Abdullah II Center for Research and Laboratory Sciences, Royal Medical Services, Amman, Jordan; 5 Laboratory Section, Medical Commission Department, Ministry of Public Health, Doha, Qatar; 6 Department of Clinical Chemistry, Faculty of Allied Health Sciences, Chulalongkorn University, Patumwan, Bangkok, 10330 Thailand; 7 Medical Technology Unit, Health Sciences Service Center, Faculty of Allied Health Sciences, Chulalongkorn University, Bangkok, Thailand; 8 Department of Transfusion Medicine and Clinical Microbiology, Faculty of Allied Health Sciences, Chulalongkorn University, Bangkok, 10330 Thailand; 9 Mathematics Program, Department of Mathematics and Statistics, College of Arts and Sciences, Qatar University, Doha, Qatar; 10 Infectious Disease Epidemiology Group, Weill Cornell Medicine-Qatar, Cornell University, Doha, Qatar; 11 Department of Population Health Sciences, Weill Cornell Medicine, Cornell University, New York, NY USA

**Keywords:** Cytomegalovirus, CMV IgG, CMV IgM, CLIA, Mindray, NovaLisa, Diseases, Immunology, Medical research, Microbiology

## Abstract

CMV serology is vital for screening at-risk populations. While IgG assays show high accuracy, IgM interpretation is challenged by assay variability and the lack of a gold standard, especially in high-prevalence settings. This study evaluates four platforms to refine diagnostic classification. Performance of Mindray (CLIA), Abbott (CMIA), Roche (ECLIA), and NovaLisa (ELISA) was assessed using 319 (IgG) and 237 (IgM) samples against a “composite silver standard” (concordance in ≥ 3 assays). IgG avidity adjudicated discordant IgM results. CMV IgG seropositivity was consistent: Mindray (71.8%), Abbott (71.5%), NovaLisa (69.0%), and Roche (67.1%), aligning with the silver standard (69.6%). IgG assays showed almost perfect agreement (κ = 0.91–0.95), with sensitivities 96.9–99.1% and specificities 90.5–98.9%. Conversely, IgM seropositivity varied significantly: Abbott (18.6%) and NovaLisa (18.1%) were significantly higher than the silver standard (5.5%; *p* < 0.01), while Mindray (8.9%) and Roche (9.7%) aligned closer. IgM agreement was only fair-to-moderate (κ = 0.34–0.58). Avidity confirmed most isolated IgM-positive results did not reflect recent infection. CMV IgG detection is robust across platforms and suitable for clinical use. However, marked IgM discordance limits its standalone utility, supporting an IgG-based diagnostic strategy supplemented by avidity testing in high-prevalence populations.

## Introduction

Cytomegalovirus (CMV) is a ubiquitous virus that establishes lifelong latency following primary infection^1–3^. Although often asymptomatic in immunocompetent individuals, CMV poses a significant risk to pregnant women, transplant recipients, and immunocompromised individuals, where primary infection or viral reactivation may cause severe disease^4–7^. Congenital CMV infection is the most common congenital viral infection worldwide and remains a leading non-genetic cause of sensorineural hearing loss and long-term neurodevelopmental impairment^8,9^.

Human infection is typically acquired through contact with infected bodily fluids—including saliva, urine, blood, genital secretions, and breast milk—; and may occur through transplacental, perinatal, or transfusion-related routes^4,10,11^. Because primary CMV infection frequently presents with non-specific or absent symptoms, laboratory-based diagnosis is essential for identifying at-risk individuals, monitoring vulnerable populations, and preventing mother-to-child transmission^12–16^.

Serological testing is central to CMV screening strategies. CMV-specific IgG reflects past exposure and long-term immunity, while IgM is traditionally interpreted as a marker of recent primary infection or viral reactivation^13,17^. However, IgM interpretation is complicated by prolonged or recurrent positivity, assay-dependent cross-reactivity, and limited specificity, making IgM unreliable as a stand-alone indicator of acute infection^16,18–20^.

Given these limitations, IgG avidity testing has become an essential confirmatory tool for distinguishing recent from past infection. Low-avidity IgG typically indicates a primary infection within the preceding 2–4 months, whereas high-avidity IgG effectively rules out recent infection even in the presence of reactive IgM. Because primary maternal infection carries the highest risk of congenital transmission, avidity testing has become integral to obstetric evaluation and several clinical management algorithms^17,21^. In diagnostic accuracy studies, avidity further improves interpretation by adjudicating discordant IgM results and refining uncertain serological profiles.

Historically, enzyme-linked immunosorbent assays (ELISAs) have been widely used for CMV serodiagnosis^21–23^, but they are limited by manual processing steps, longer turnaround times, and variable analytical performance. Chemiluminescent immunoassays (CLIAs), including CMIA and ECLIA technologies, have gained prominence due to improved sensitivity, automation, and suitability for high-throughput testing^24,25^. Among these, the Abbott ARCHITECT (CMIA) and Roche Cobas (ECLIA) platforms are the most widely adopted globally, while the Mindray CL-series, a newer CLIA platform, has fewer large-scale comparative evaluations^26^.

Globally, CMV seropositivity varies considerably—from approximately 40% in high-income countries to more than 80–90% in parts of Africa, Asia, and the Middle East—reflecting differences in socioeconomic factors, population density, and early childhood exposures^27–29^. In high-prevalence settings such as the Middle East, diagnostic interpretation becomes increasingly complex: isolated IgM reactivity rarely indicates true primary infection, while missing seronegative individuals may have important clinical consequences in pregnancy, transplantation, and immunosuppression^21^. Misclassification may lead to unnecessary follow-up and anxiety in pregnant women with false-positive IgM, or inappropriate donor–recipient assignment in transplant programs^30^. These epidemiological dynamics underscore the need for precise and reliable CMV serology, particularly when assay variability may influence clinical decision-making.

A major challenge in CMV serodiagnosis is the absence of a universally accepted gold standard. Reliance on a single assay can lead to misclassification due to antigen selection, cut-off calibration, and assay-specific differences in analytical sensitivity. Traditional accuracy studies are limited by the reference method used. To address this, contemporary evaluations often employ composite reference frameworks, or “silver standards,” based on concordant results from three or more immune assays^31–33^. This approach reduces bias and provides a more reliable assessment of true infection status, particularly for CMV, where IgM discordance is common. Using a composite silver standard in this study ensures a more balanced evaluation of assay performance without over-relying on any single platform.

Accordingly, this study aims to systematically compare the diagnostic performance of four widely used CMV antibody assays, three CLIA platforms (Mindray, Abbott, Roche) and one ELISA (NovaLisa), for both IgG and IgM detection, using a composite silver standard. Importantly, we also incorporate CMV IgG avidity testing to adjudicate discordant or ambiguous IgM patterns, refine infection classification, and strengthen the accuracy of assay benchmarking. Through this combined approach, we evaluate inter-assay agreement, quantify variability across platforms, and contrast “positive by any assay” with “positive by all assays” to capture the full spectrum of diagnostic discrepancies.

## Methodology

### Study design

#### Ethical compliance

Ethical approval for this study was obtained from the Institutional Review Board at Qatar University (QU-IRB 2014-E/23).

#### Study population

A total of 319 serum samples were tested for IgG. Of these, 237 samples were also tested for IgM. Of the 237 IgM-tested samples, 82 samples underwent IgG avidity testing to assess the likelihood of recent primary infection. This sample size was strategically selected to exceed the minimum requirements of CLSI EP12-A2, which typically recommends at least 50 positive and 50 negative specimens for qualitative method comparisons^34^. By utilizing a larger cohort (*n* > 200 for each marker), this study ensured high statistical power and narrow 95% confidence intervals for sensitivity and specificity estimates. Given the source of the specimens from a major tertiary reference laboratory within the Jordanian Royal Medical Services, the cohort represents a diverse clinical referral population, primarily comprised of individuals undergoing routine or high-risk serological screening, including antenatal patients, transplant candidates, and immunocompromised individuals for whom CMV status is clinically critical. The inclusion of this high volume of samples allowed for a comprehensive evaluation of the “gray zone” near assay cut-offs, where diagnostic discordance is most frequent.

The study design followed a multi-platform comparative approach to verify the performance of both established, FDA-approved CLIA and newer diagnostic technologies^26^. To ensure analytical reliability and sample commutability, all procedures were conducted in alignment with ISO 15189:2022 and CAP accreditation standards^35,36^. Pre-analytical integrity was prioritized by aliquoting and storing all specimens at −80 °C to eliminate the risk of antibody degradation from freeze-thaw cycles, consistent with IFCC recommendations. This robust framework provides a rigorous benchmark for comparing the clinical utility of the newer Mindray platform against globally recognized industry standards.

### Diagnostic assays

#### Roche Cobas 6000 e Module (ECLIA)

The Roche Cobas 6000 e module, based on electrochemiluminescence immunoassay (ECLIA) technology, was used for the detection of CMV IgG and IgM antibodies^37^. Assays were performed on a fully automated platform according to the manufacturer’s instructions. Hemolyzed, lipemic, and icteric samples were excluded. Prior to testing, the analyzer was calibrated using Roche-provided CMV calibrators. Both low and high CMV IgG/IgM controls were included in each run to ensure assay validity. The analytical steps included incubation of serum with CMV-specific antigen-coated microparticles, addition of ruthenium-labeled conjugates for signal generation, magnetic bead-based separation and washing, followed by electrochemiluminescence detection using a photomultiplier.

####  Mindray CL-series analyzer (CLIA)

The Mindray CMV IgG and IgM assays (Shenzhen Mindray Bio-Medical Electronics Co., Ltd., China) are CLIAs designed for the qualitative detection of CMV-specific IgG and IgM antibodies in human serum and plasma. All 237 serum samples were processed with the exclusion of hemolyzed, lipemic, or icteric samples. Both assays were performed on the fully automated Mindray CL-900i platform, following the manufacturer’s instructions. For CMV IgG, the system utilizes CMV antigen-coated paramagnetic microparticles to bind target antibodies, followed by incubation with alkaline phosphatase-labeled anti-human IgG. After a wash step, a chemiluminescent substrate is added and catalyzed, generating a light signal measured in relative light units (RLUs). CMV IgM detection follows a similar protocol, with microparticles coated with CMV antigens and the conjugate composed of alkaline phosphatase-labeled anti-human IgM. The intensity of the chemiluminescent signal correlates with antibody concentration and is compared to an onboard-calculated cutoff RLU. Index values were calculated as sample RLU divided by cutoff RLU, and results were interpreted as reactive (≥ 1.10), nonreactive (< 0.90), or indeterminate (0.90–1.10). Each run included appropriate positive and negative controls, and testing was performed in accordance with routine instrument calibration and quality control standards.

#### Abbott architect i system (CMIA)

The ARCHITECT CMV IgG and IgM assays (Abbott Diagnostics, Ireland) are chemiluminescent microparticle immunoassays (CMIA) performed on the fully automated ARCHITECT iSystem for the qualitative detection of CMV-specific IgG and IgM antibodies in human serum and plasma^38,39^. A total of 181 serum samples were tested using this platform. Both assays were conducted as per the manufacturer’s instructions using the Chemiflex protocol. For CMV IgG, the assay involved a two-step procedure where serum, assay diluent, and CMV (strain AD169) lysate-coated paramagnetic microparticles were incubated, allowing specific IgG binding. Following a wash step, a murine acridinium-labeled anti-human IgG conjugate was added. A chemiluminescent reaction was triggered, and relative light units (RLUs) were measured. The signal was compared to a pre-established calibrator to determine positivity. Specimens with values ≥ 6.0 AU/mL were considered reactive. For CMV IgM, a similar two-step CMIA format was used with antigen-coated microparticles and murine acridinium-labeled anti-human IgM conjugate. The assay included goat anti-human IgG in the diluent to minimize rheumatoid factor interference. Samples were interpreted based on an index value, with results ≥ 1.00 considered reactive. Both assays included calibrators and internal controls and were validated by daily quality control as per the manufacturer’s recommendations. All samples were processed, analyzed, and interpreted automatically by the ARCHITECT iSystem.

#### NovaLisa gold standard diagnostics (ELISA)

Serum samples were tested for CMV-specific IgG and IgM antibodies using the NovaLisa CMV IgG and IgM ELISA kits (Gold Standard Diagnostics, Frankfurt, Germany), according to the respective manufacturer’s protocols^40,41^. Both assays are based on an indirect ELISA principle, wherein microplates are coated with CMV antigens that capture specific antibodies present in the sample. After the initial incubation with diluted serum (1:100 in diluent for IgG; undiluted or as specified for IgM), unbound components were removed by washing with a diluted phosphate-buffered solution. Horseradish peroxidase (HRP)-conjugated anti-human IgG or IgM antibodies were then added, forming immune complexes with the captured antibodies. The addition of tetramethylbenzidine (TMB) substrate enabled enzymatic color development, resulting in a blue color. After a timed incubation (15 min for IgG, as specified for IgM), the reaction stopped using sulfuric acid, resulting in a color change to yellow. Absorbance was measured at 450 nm (with optional reference at 620 nm) within 30 min using a microplate reader. Run validation criteria were strictly followed for both assays, including absorbance thresholds for substrate blank, negative, cutoff, and positive controls. Results were calculated based on the respective cutoff formulas provided by the manufacturer and interpreted as negative, equivocal, or positive based on optical density values relative to the cutoff.

##### IgG avidity testing (NovaLisa CMV IgG ELISA)

CMV IgG avidity was assessed using the NovaLisa CMV IgG ELISA kit according to the manufacturer’s instructions with the addition of urea as a chaotropic dissociation step. Briefly, IgG-positive serum samples were tested in duplicate, with one well treated with a urea-containing dissociation solution and the paired well serving as a control. The avidity index (AI) was calculated as the ratio of absorbance in the urea-treated well to the untreated well, multiplied by 100. Low AI values indicate recent primary CMV infection, whereas high AI values reflect past infection and mature IgG responses; intermediate results were interpreted as equivocal according to manufacturer guidelines.

### Statistical analysis

Demographic data was summarized using Microsoft Excel. Diagnostic performance was assessed by comparing the results of Mindray CLIA, Abbott CLIA, and NovaLisa ELISA to the Roche CLIA assay, which served as the reference method. Additionally, comparisons were made: (i) between all assays (Mindray, Abbott, Roche, and NovaLisa), (ii) against the composite silver standard, and (iii) across the assays to evaluate their concordance and diagnostic accuracy. For both IgG and IgM assays, equivocal and borderline results were classified as positive and included in all statistical analyses; no indeterminate values were excluded, acknowledging their potential clinical relevance and avoiding exclusion-related bias. Diagnostic agreement was evaluated using standard metrics: sensitivity, specificity, Positive Predictive Value (PPV), Negative Predictive Value (NPV), accuracy, and Cohen’s kappa coefficient with 95% confidence intervals. Kappa values were interpreted as follows: ≤0 (no agreement), 0.01–0.20 (slight), 0.21–0.40 (fair), 0.41–0.60 (moderate), 0.61–0.74 (good), 0.75–0.80 (substantial), and 0.81–1.00 (almost perfect agreement)^42–46^. All statistical calculations, including confidence intervals, Chi-square testing for association, and agreement analyses, were performed using GraphPad Prism (version 10.4.1, GraphPad Software, San Diego, CA, USA). Where appropriate, sensitivity, specificity, PPV, and NPV were calculated with 95% confidence intervals using Prism’s built-in diagnostic test evaluation tools.

#### Diagnostic performance metrics and evaluation

Diagnostic performance was assessed using two complementary approaches. First, because there is no universally accepted serologic gold standard for CMV, we defined a composite silver standard to approximate true status: samples were classified silver-standard positive if reactive in ≥ 3 of the evaluated immunoassays (Mindray CLIA, Abbott CLIA, Roche ECLIA, NovaLisa ELISA) and silver-standard negative if non-reactive in all assays; mixed patterns not meeting either criterion were considered indeterminate and were excluded from silver-standard accuracy calculations. Second, we summarized seropositive using prespecified composite rules to contextualize assay discordance in this cohort: (i) Positive by Any Assay (reactive on ≥ 1 platform; upper-bound, highly sensitive estimate) and (ii) Positive by All Assays (reactive on all four platforms; lower-bound, highly specific estimate). These composites were treated as descriptive sensitivity analyses and were not used for inferential comparisons against their component assays to avoid circularity. For per-assay diagnostic accuracy vs. the silver standard (primary analysis), we computed sensitivity, specificity, PPV, NPV, accuracy and Cohen’s κ with 95% CI. Seropositive proportions are presented with 95% Wilson confidence intervals.

## Results

This study assessed CMV IgG and IgM seropositive using four diagnostic platforms: Mindray (CLIA), Roche (ECLIA), NovaLisa (ELISA), and Abbott (CLIA). Seropositivity estimates were determined for each assay, and statistical comparisons were performed to evaluate inter-assay agreement and potential variability in detection rates. Separate analyses were conducted for IgG and IgM to reflect their differing clinical relevance and to identify any assay-specific performance trends.

### Kit evaluation

**Fig. 1:**
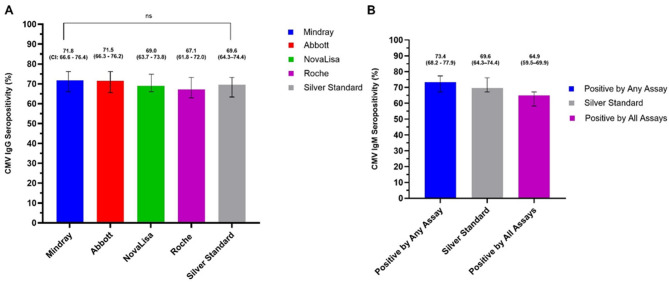
CMV IgG seropositivity across four commercial assays and composite definitions.** (A)** Bars represent the percentage of CMV IgG–positive samples detected by Mindray, Abbott, NovaLisa, Roche, and the Silver Standard reference in the same cohort (*n* = 319). Error bars denote 95% confidence intervals and reflect uncertainty in the estimated seropositivity rather than variability of individual measurements; exact seropositivity estimates and confidence intervals are shown above each bar for all assays: Mindray, Abbott, NovaLisa, Roche, and Silver Standard. Chi-square tests comparing each assay to the Silver Standard showed no statistically significant differences (all *p* > 0.05). **(B)** Composite IgG seropositivity estimates derived from the same dataset are shown for contextual comparison: Positive by Any Assay, Silver Standard, and Positive by All Assays. These composite definitions provide upper-bound, reference, and lower-bound estimates of CMV IgG seropositivity within the study population.

The comparison of CMV IgG seropositivity across platforms revealed closely aligned estimates with minimal dispersion (Fig. 1). Mindray demonstrated the highest seropositivity at 71.8% (95% CI: 66.6–76.4), followed closely by Abbott at 71.5% (95% CI: 65.3–76.2), NovaLisa at 69.0% (95% CI: 63.7–73.8), and Roche at 67.1% (95% CI: 61.8–72.0). The Silver Standard reference yielded a comparable estimate of 69.6% (95% CI: 64.3–74.4), falling within the range of all individual assays (Fig. 1A). When composite definitions were applied, the “positive by any assay” criterion provided an upper-bound estimate of 73.4% (95% CI: 68.2–77.9), whereas the more stringent “positive by all assays” definition resulted in a lower-bound estimate of 64.9% (95% CI: 59.5–69.9) (Fig. 1B). Chi-square testing showed no statistically significant differences between any individual assay and the Silver Standard (all *p* > 0.05), indicating high inter-assay concordance and limited variability in CMV IgG seropositivity estimates within this cohort.

**Fig. 2:**
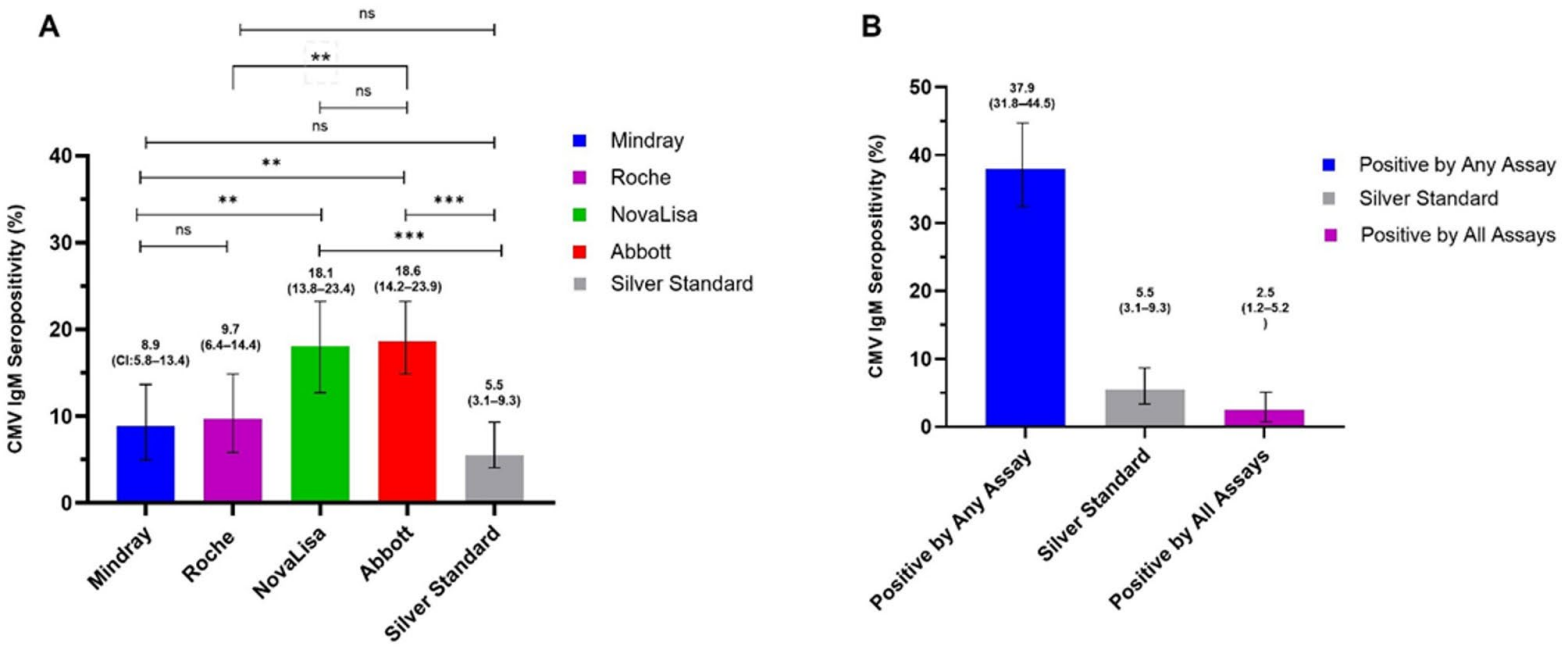
**(A)** Bars represent the percentage of CMV IgM–positive samples detected by Mindray, Roche, NovaLisa, Abbott, and the Silver Standard reference (defined as reactivity on ≥ 3 of 4 assays) in the same cohort (*n* = 237). Error bars denote 95% confidence intervals and reflect uncertainty in the estimated seroprevalence rather than variability of individual measurements; exact seroprevalence estimates and confidence intervals are shown above each bar for all assays. Chi-square tests comparing each assay to the Silver Standard showed no statistically significant differences for Mindray and Roche (ns), while NovaLisa (*p < 0.05) and Abbott (**p < 0.01) demonstrated significantly higher IgM seroprevalence. **(B)** Composite IgM seroprevalence estimates derived from the same dataset are shown for contextual comparison: Positive by Any Assay, Silver Standard, and Positive by All Assays. These composite definitions provide upper-bound, reference, and lower-bound estimates of CMV IgM seroprevalence within the study population.

CMV IgM seropositivity differed substantially across the evaluated platforms (Fig. 2A). Mindray and Roche yielded lower and comparable IgM positivity rates at 8.9% (95% CI: 5.8–13.4) and 9.7% (95% CI: 6.4–14.4), respectively, both of which were not significantly different from the composite Silver Standard estimate of 5.5% (95% CI: 3.1–9.3; independent two-proportion χ², ns). In contrast, NovaLisa and Abbott produced significantly higher IgM seropositivity estimates—18.1% (95% CI: 13.8–23.4) and 18.6% (95% CI: 14.2–23.9), respectively—compared with the Silver Standard (*p* < 0.01 and *p* < 0.001). Composite analyses further highlighted the extent of inter-assay discordance (Fig. 2B). The “Positive by Any Assay” definition yielded a markedly higher upper-bound estimate of IgM seropositivity at 37.9% (95% CI: 31.8–44.5), whereas the stringent “Positive by All Assays” criterion resulted in a low lower-bound estimate of 2.5% (95% CI: 1.2–5.2). Together, these findings indicate that isolated CMV IgM reactivity is frequent and highly assay-dependent, while concordant IgM positivity across platforms is uncommon in this cohort.

### Diagnostic Performance and inter-assay agreement of CMV IgG assays

Table 1 presents a comprehensive comparison of the diagnostic performance of CMV IgG assays across all pairwise evaluations, demonstrating consistently high agreement among the tested platforms (*n* = 319). Across comparisons, sensitivity values were uniformly high, generally exceeding 90%, while specificity remained robust, most often above 88%. Positive and negative predictive values were similarly strong, indicating reliable identification of both CMV IgG–positive and –negative samples. Overall agreement was almost perfect in all comparisons, with Cohen’s kappa values ranging from 0.84 to 0.96, all interpreted as almost perfect agreement. When referenced against other platforms, Abbott and Mindray showed particularly strong concordance, achieving kappa values up to 0.96 and maintaining balanced sensitivity and specificity profiles. Roche also demonstrated almost perfect agreement with all comparator assays, with kappa values consistently above 0.87. Although NovaLisa showed slightly lower specificity or NPV in certain pairings, its overall diagnostic performance remained high, with kappa values above 0.87, supporting its reliability. Notably, the “Positive by Any Assay” approach yielded the highest concordance across reference comparisons, with sensitivities approaching 98–98.1%, specificities of 98.8%, and kappa values up to 0.95. Collectively, these findings confirm the strong inter-assay reliability and clinical utility of all evaluated CMV IgG detection platforms, with almost perfect agreement maintained across individual and composite reference standards.

**Table 1:** Comparative diagnostic performance of CMV IgG Assays: Sensitivity, Specificity, PPV, NPV, Accuracy, and Cohen’s Kappa (*n* = 319).

Reference Kit	Compared Kit	Sensitivity (CI: 95%)	Specificity (CI: 95%)	PPV (CI: 95%)	NPV (CI: 95%)	Kappa	Kappa Interpretation
**Roche**	Positive by Any Assay	91.5 (87.2–94.4)	98.8 (93.7–99.9)	99.5 (97.4–100.0)	81.0 (72.4–87.3)	0.843 (0.780–0.907)	Almost perfect
	Mindray	92.6 (88.4–95.3)	97.8 (92.3–99.6)	99.1 (96.7–99.8)	83.8 (75.6–89.6)	0.860 (0.799–0.920)	Almost perfect
	Abbott	93.4 (89.4–96.0)	98.9 (94.0–100.0)	99.5 (97.4–100.0)	85.7 (77.8–91.2)	0.882 (0.826–0.938)	Almost perfect
	NovaLisa	94.6 (90.7–96.9)	93.9 (87.4–97.2)	97.2 (94.0–98.7)	88.6 (81.1–93.3)	0.870 (0.812–0.928)	Almost perfect
**Mindray**	Positive by Any Assay	97.9 (95.1–99.1)	98.8 (93.7–99.9)	99.6 (97.6–100.0)	94.4 (87.7–97.6)	0.952 (0.915–0.990)	Almost perfect
	Roche	99.1 (96.7–99.8)	83.8 (75.6–89.6)	92.6 (88.4–95.3)	97.8 (92.3–99.6)	0.860 (0.799–0.920)	Almost perfect
	Abbott	97.8 (92.3–99.6)	96.7 (90.7–99.1)	96.7 (90.8–99.1)	97.8 (92.3–99.6)	0.961 (0.927–0.994)	Almost perfect
	NovaLisa	99.1 (96.7–99.8)	87.9 (80.0–92.9)	94.8 (91.1–97.0)	97.8 (92.2–99.6)	0.894 (0.840–0.948)	Almost perfect
**Abbott**	Positive by Any Assay	97.4 (94.5–98.8)	98.8 (93.7–99.9)	99.6 (97.6–100.0)	93.4 (86.4–97.0)	0.945 (0.905–0.985)	Almost perfect
	Roche	99.5 (97.4–100.0)	85.7 (77.8–91.2)	93.4 (89.4–96.0)	98.9 (94.0–99.9)	0.882 (0.826–0.938)	Almost perfect
	Mindray	98.7 (96.2–99.6)	97.8 (92.3–99.6)	99.1 (96.9–99.8)	96.7 (90.8–99.1)	0.961 (0.927–0.994)	Almost perfect
	NovaLisa	99.1 (96.8–99.8)	89.9 (82.4–94.4)	95.6 (92.1–97.6)	97.8 (92.3–99.6)	0.910 (0.860–0.959)	Almost perfect
**NovaLisa**	Positive by Any Assay	94.0 (90.2–96.4)	98.8 (93.7–99.9)	99.6 (97.5–100.0)	85.9 (77.7–91.4)	0.886 (0.830–0.942)	Almost perfect
	Roche	97.2 (94.0–98.7)	88.6 (81.1–93.3)	94.6 (90.7–96.9)	93.9 (87.4–97.2)	0.870 (0.812–0.928)	Almost perfect
	Mindray	94.8 (91.1–97.0)	96.7 (90.7–99.1)	98.6 (96.1–99.6)	87.9 (80.0–92.9)	0.887 (0.831–0.942	Almost perfect
	Abbott	95.6 (92.1–97.6)	97.8 (92.3–99.6)	99.1 (96.8–99.8)	89.9 (82.4–94.4)	0.910 (0.860–0.959)	Almost perfect

Table 2 summarizes the diagnostic performance and agreement of the evaluated CMV IgG assays using the composite silver standard as the reference. Overall, all platforms demonstrated high diagnostic accuracy and almost perfect inter-assay agreement, with consistently strong sensitivity, specificity, PPV, and NPV values. Among the evaluated assays, Roche CLIA showed the highest sensitivity at 99.1% (95% CI: 96.7–99.8), followed closely by NovaLisa ELISA at 98.6% (96.1–99.6), Abbott CLIA at 97.4% (94.4–98.8), and Mindray CLIA at 96.9% (93.8–98.5). Specificity remained high across assays, ranging from 90.5% (83.4–94.7) for Roche to 98.9% (94.0–99.9) for both Mindray and Abbott. PPVs were uniformly high (95.5%–99.6%), while NPVs ranged from 92.8% to 97.9%, indicating reliable exclusion of CMV IgG negativity. Agreement analysis further confirmed almost perfect concordance between all assays and the silver standard, with Cohen’s kappa values ranging from 0.913 to 0.947, underscoring the robust and consistent performance of CMV IgG testing across platforms.

**Table 2:** Diagnostic and agreement metrics for CMV IgG assays (*n* = 319).

Reference Kit	Compared Kit	Sensitivity (CI: 95%)	Specificity (CI: 95%)	PPV (CI: 95%)	NPV (CI: 95%)	Kappa	Kappa Interpretation
**Silver Standard**	Mindray	96.9 (93.8–98.5)	98.9 (94.0–99.9)	99.6 (97.5–100.0)	92.8 (85.9–96.5)	0.939 (0.898–0.980)	Almost perfect
	Abbott	97.4 (94.4–98.8)	98.9 (94.1–99.9)	99.6 (97.5–100.0)	93.8 (87.2–97.1)	0.947 (0.908–0.985)	Almost perfect
	NovaLisa	98.6 (96.1–99.6)	94.9 (88.7–97.8)	97.8 (94.8–99.0)	96.9 (91.3–99.2)	0.941 (0.900–0.981)	Almost perfect
	Roche	99.1 (96.7–99.8)	90.5 (83.4–94.7)	95.5 (91.9–97.5)	97.9 (92.8–99.6)	0.913 (0.865–0.961)	Almost perfect

### Diagnostic performance and inter-assay agreement of CMV IgM assays

Table 3 presents the pairwise comparative diagnostic performance of CMV IgM assays across platforms (*n* = 237). When Roche was used as the reference, sensitivity ranged from 25.6% (95% CI: 17.7–35.4) for the “positive by any assay” definition to 47.6% (28.3–67.6) for Mindray, while specificity remained high across comparisons (93.3%–94.3%). Corresponding PPV values ranged from 43.5% to 95.8%, and NPV values from 68.7% to 94.9%. Using Mindray as the reference, sensitivities varied between 23.3% (15.8–33.1) for the “positive by any assay” rule and 43.5% (25.6–63.2) for Roche, with specificity consistently high (93.3%–96.9%). PPV values ranged from 47.6% to 95.5%, and NPV from 68.1% to 94.0%. When Abbott served as the reference, sensitivity ranged from 48.9% (38.8–59.1) for the “positive by any assay” definition to 52.2% (33.0–70.8) for Roche, while specificity ranged from 86.6% to 93.3%. PPV values varied between 27.9% and 97.8%, and NPV between 75.8% and 94.9%. Using NovaLisa as the reference, sensitivity ranged from 47.8% (37.8–58.0) for the “positive by any assay” definition to 52.2% (33.0–70.8) for Roche, with specificity between 84.7% and 99.3%. PPV values ranged from 23.3% to 97.7%, and NPV from 75.8% to 94.3%.

Inter-assay agreement analyses demonstrated predominantly fair concordance across pairwise comparisons. When Roche was used as the reference, Cohen’s kappa values ranged from 0.290 to 0.398, indicating fair agreement across all comparisons. Using Mindray as the reference, agreement remained fair, with kappa values ranging from 0.219 to 0.398. When Abbott served as the reference, agreement ranged from fair to moderate, with kappa values between 0.282 and 0.534, including moderate agreement for the “positive by any assay” definition (κ = 0.534). Using NovaLisa as the reference, kappa values ranged from 0.219 to 0.542, reflecting fair to moderate agreement across comparisons.

**Table 3:** Comparative diagnostic performance of CMV IgM assays: sensitivity, specificity, PPV, NPV, accuracy, and Cohen’s Kappa (*n* = 237).

Reference Kit	Compared Kit	Sensitivity (CI: 95%)	Specificity (CI: 95%)	PPV (CI: 95%)	NPV (CI: 95%)	Kappa	Kappa Interpretation
**Roche**	Positive by Any Assay	25.6 (17.7–35.4)	99.3 (96.3–100.0)	95.8 (79.8–99.8)	68.7 (62.2–74.5)	0.290 (0.187–0.393)	Fair
	Mindray	47.6 (28.3–67.6)	94.0 (90.0–96.5)	43.5 (25.6–63.2)	94.9 (91.0–97.1)	0.398 (0.203–0.594)	Fair
	Abbott	34.3 (20.8–50.9)	94.3 (90.1–96.8)	52.2 (33.0–70.8)	88.8 (83.7–92.4)	0.332 (0.160–0.504)	Fair
	NovaLisa	27.9 (16.8–42.7)	94.3 (90.1–96.8)	52.2 (33.0–70.8)	85.5 (80.2–89.6)	0.271 (0.113–0.429)	Fair
**Mindray**	Positive by Any Assay	23.3 (15.8–33.1)	99.3 (96.3–100.0)	95.5 (78.2–99.8)	68.1 (61.6–73.9)	0.265 (0.165–0.366)	Fair
	Roche	43.5 (25.6–63.2)	94.9 (91.0–97.1)	47.6 (28.3–67.6)	94.0 (90.0–96.5)	0.398 (0.203–0.594)	Fair
	Abbott	34.1 (21.9–48.9)	96.9 (93.4–98.6)	71.4 (50.0–86.2)	86.6 (81.4–90.5)	0.388 (0.230–0.545)	Fair
	NovaLisa	23.3 (13.2–37.7)	94.3 (90.1–96.8)	47.6 (28.3–67.6)	84.7 (79.3–88.9)	0.219 (0.064–0.374)	Fair
**Abbott**	Positive by Any Assay	48.9 (38.8–59.1)	99.3 (96.3–100.0)	97.8 (88.4–99.9)	76.2 (69.7–81.6)	0.534 (0.428–0.641)	Moderate
	Roche	52.2 (33.0–70.8)	85.1 (79.7–89.2)	27.3 (16.4–41.9)	94.3 (90.1–96.8)	0.332 (0.160–0.504)	Fair
	Mindray	71.4 (50.0–86.2)	86.6 (81.4–90.5)	34.1 (21.9–48.9)	96.9 (93.4–98.6)	0.388 (0.230–0.545)	Fair
	NovaLisa	41.9 (28.4–56.7)	86.6 (81.1–90.7)	40.9 (27.7–55.6)	87.1 (81.6–91.1)	0.282 (0.132–0.431)	Fair
**NovaLisa**	Positive by Any Assay	47.8 (37.8–58.0)	99.3 (96.3–100.0)	97.7 (88.2–99.9)	75.8 (69.3–81.3)	0.542 (0.434–0.650)	Moderate
	Roche	52.2 (33.0–70.8)	85.5 (80.2–89.6)	27.9 (16.8–42.7)	94.3 (90.1–96.8)	0.271 (0.113–0.429)	Fair
	Mindray	47.6 (28.3–67.6)	84.7 (79.3–88.9)	23.3 (13.2–37.7)	94.3 (90.1–96.8)	0.219 (0.064–0.374)	Fair
	Abbott	40.9 (27.7–55.6)	87.1 (81.6–91.1)	41.9 (28.4–56.7)	86.6 (81.1–90.7)	0.282 (0.132–0.431)	Fair

Table 4 summarizes the diagnostic performance and agreement of the evaluated CMV IgM assays using the composite silver standard as the reference. Across platforms, IgM detection was characterized by modest sensitivity and consistently high specificity. Sensitivity ranged from 25.6% (95% CI: 14.9–40.2) for NovaLisa ELISA to 47.8% (29.2–67.0) for Roche CLIA and 47.6% (28.3–67.6) for Mindray CLIA, while Abbott CLIA demonstrated a sensitivity of 29.6% (18.2–44.2). Specificity was uniformly high, exceeding 98% for all assays and reaching 99.5% (97.1–100.0) for Abbott CLIA. PPVs ranged from 76.9% to 92.9%, and NPVs remained high across platforms (85.7%–95.1%). Agreement analysis showed fair to moderate concordance with the silver standard, with Cohen’s kappa values ranging from 0.337 to 0.581. Moderate agreement was observed for Mindray and Roche (κ = 0.558 and 0.581, respectively), whereas Abbott and NovaLisa demonstrated fair agreement (κ = 0.394 and 0.337).

**Table 4:** Diagnostic and agreement metrics for CMV IgM assays (*n* = 237).

Reference Kit	Compared Kit	Sensitivity (CI: 95%)	Specificity (CI: 95%)	PPV (CI: 95%)	NPV (CI: 95%)	Kappa	Kappa Interpretation
**Silver Standard**	Mindray	47.6 (28.3–67.6)	98.6 (96.0–99.6)	76.9 (49.7–91.8)	95.1 (91.4–97.2)	0.558 (0.353–0.763)	Moderate
	Abbott	29.6 (18.2–44.2)	99.5 (97.1–100.0)	92.9 (68.5–99.6)	86.2 (81.0–90.1)	0.394 (0.236–0.551)	Fair
	NovaLisa	25.6 (14.9–40.2)	99.0 (96.3–99.8)	84.6 (57.8–97.3)	85.7 (80.5–89.7)	0.337 (0.178–0.495)	Fair
	Roche	47.8 (29.2–67.0)	99.1 (96.7–99.8)	84.6 (57.8–97.3)	94.6 (90.9–96.9)	0.581 (0.386–0.777)	Moderate

### IgG avidity findings among IgM-positive samples

**Fig. 3:**
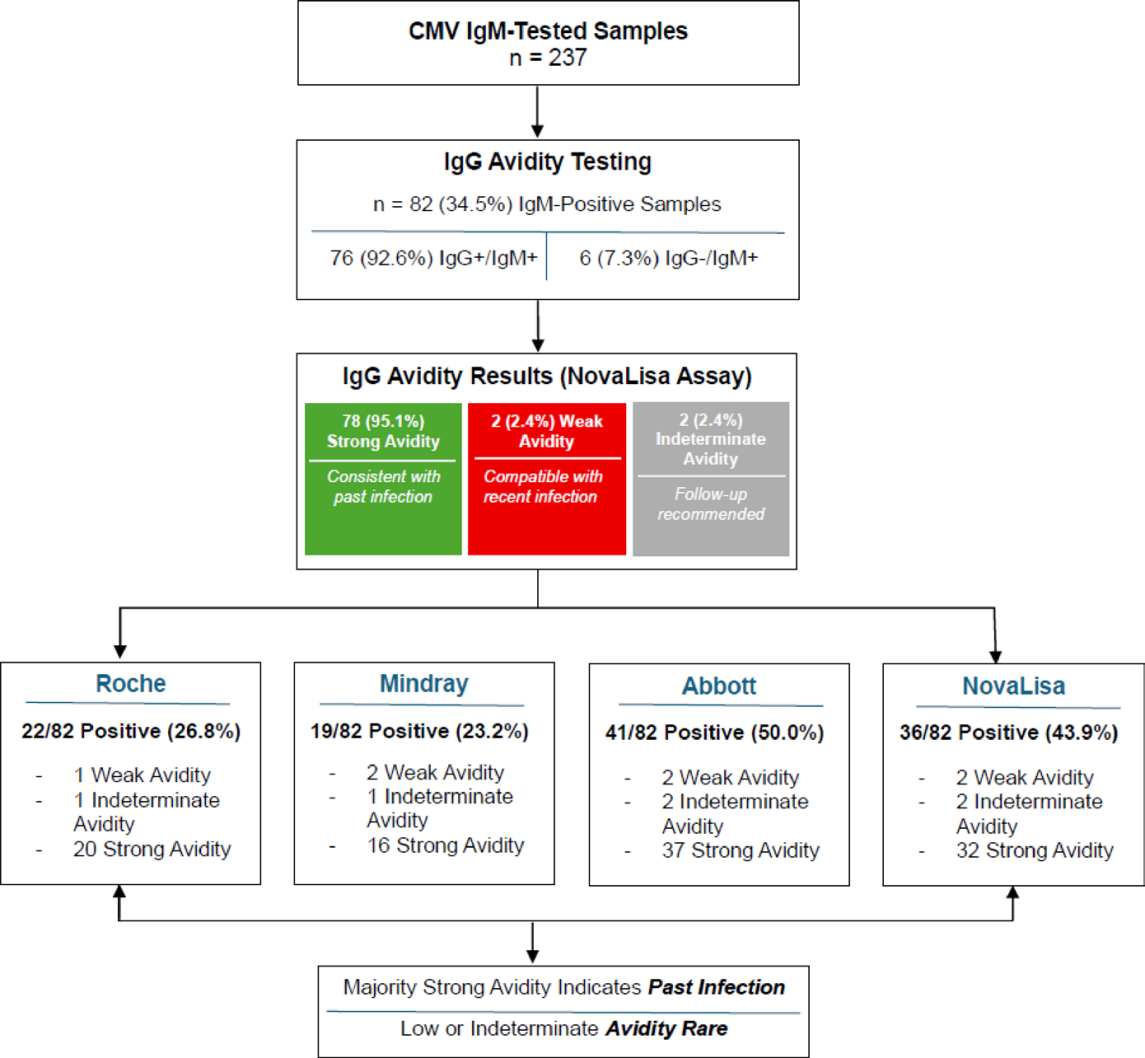
Flowchart depicting the selection of CMV IgM-tested samples, reflex IgG avidity testing among IgM-positive samples, and assay-specific IgM positivity with corresponding IgG avidity classifications. The diagram outlines the progression from initial CMV IgM testing to IgG avidity assessment using the NovaLisa assay and presents the distribution of IgM-positive samples detected by Roche ECLIA, Mindray CLIA, Abbott CMIA, and NovaLisa ELISA within the avidity-tested subset.

IgG avidity testing was performed on a subset of IgM-positive samples to evaluate the likelihood of recent primary CMV infection. A total of 237 IgM-tested samples were considered, of which 82 (34.5%) IgM-positive samples underwent reflex IgG avidity testing. Among these, 76 samples (92.6%) were IgG-positive/IgM-positive, while 6 samples (7.3%) were IgG-negative/IgM-positive. Using the NovaLisa IgG avidity assay, 78 of the 82 tested samples (95.1%) demonstrated strong IgG avidity, consistent with past CMV infection. Only two samples (2.4%) showed weak avidity, compatible with recent primary infection, and two samples (2.4%) exhibited indeterminate avidity, for which follow-up testing was recommended (Fig. 3).

Stratification by assay among the avidity-tested subset (*n* = 82) showed that IgM positivity varied across platforms, with Abbott CMIA detecting the highest proportion of IgM-positive samples (41/82, 50.0%), followed by NovaLisa ELISA (36/82, 43.9%), Roche ECLIA (22/82, 26.8%), and Mindray CLIA (19/82, 23.2%). Despite these differences in IgM detection frequency, strong IgG avidity predominated across all assays, whereas weak or indeterminate avidity profiles were uncommon. Overall, the flow-chart-based analysis demonstrates that the vast majority of IgM-positive samples subjected to avidity testing were consistent with past rather than recent CMV infection, and that low or indeterminate IgG avidity was rare across all platforms. These findings highlight the importance of IgG avidity testing as a reflex tool for the interpretation of CMV IgM reactivity and indicate that higher IgM detection rates do not translate into increased identification of recent primary infection.

##  Discussion

This study is the first to comprehensively evaluate the diagnostic performance of the Mindray CMV assay in comparison with two FDA-approved platforms (Roche and Abbott) and the widely used ELISA-based NovaLisa assay, using a composite silver standard framework in a high-prevalence setting. Because no universally accepted serologic gold standard exists for CMV—particularly for IgM—composite, concordance-based definitions are increasingly used to reduce reference-method bias and provide a more balanced approximation of true serostatus^32,33^. Within this framework, Mindray demonstrated performance comparable to widely deployed platforms in our study, suggesting its potential suitability for routine laboratory use in high-prevalence settings.

Across all evaluated platforms, CMV IgG assays demonstrated consistently strong diagnostic performance. High sensitivity, specificity, predictive values, and substantial to near-perfect inter-assay agreement were observed in both pairwise comparisons and silver standard analyses. These findings are consistent with prior reports demonstrating robust IgG detection using both CLIA and ELISA technologies^47,48^. Similar performance has been documented for other automated CLIA systems, which have shown excellent analytical precision, linearity, and concordance with ELISA-based methods^49,50^. Collectively, these results confirm that CMV IgG detection is reliable and reproducible, which supports its use for routine clinical screening and epidemiological surveillance, particularly in high-prevalence populations.

Although overall IgG agreement was excellent across all evaluated platforms, modest assay-dependent discordance was observed. This variability likely reflects differences in antigen composition, signal calibration, and manufacturer-defined cutoffs, factors known to influence qualitative IgG classification across ELISA, CLIA, and ECLIA platforms. Roche assays failed to detect a subset of CMV IgG-positive samples identified by other platforms, a pattern previously reported in comparative evaluations of ECLIA and CLIA methods and attributed to differences in antigen composition and assay-specific cut-off calibration that can influence analytical sensitivity^51^. In contrast, CLIA platforms showed superior analytical consistency and agreement, reflecting the advantages of automation, standardized workflows, and reduced operator dependency. Consistent with these findings, previous comparative evaluations have shown that CLIA-based platforms generally outperform ELISA assays in terms of specificity, accuracy, and operational efficiency, particularly in high-throughput laboratory settings^52^.

In contrast to IgG, CMV IgM assays showed significant variability and fair-to-moderate inter-assay concordance, indicating that performance may be influenced by assay-dependent factors. Across pairwise comparisons and silver standard analyses, sensitivity was modest and inconsistent, while agreement metrics predominantly indicated fair concordance. These findings mirror earlier reports describing the inherent limitations of CMV IgM testing, including assay-dependent cut-off variability, transient antibody expression, and limited reproducibility^16,24,53^. Abbott detected more IgM-positive samples than Mindray and Roche (*p* < 0.01), suggesting increased analytical sensitivity. However, this was associated with lower agreement and reduced PPV, highlighting the need for confirmatory testing to mitigate false-positive results. Similar patterns of increased IgM detection without improved diagnostic concordance have been attributed to broader antigen reactivity or lower positivity thresholds^24,54^. As a result, Abbott’s higher IgM reactivity reduced overall specificity unless supported by confirmatory IgG avidity testing or optimized cutoffs^21,54^, reinforcing that CMV IgM is insufficiently reproducible as a stand-alone marker of recent infection in high-prevalence settings.

Composite analyses further clarified the contrasting reliability of IgG and IgM detection across platforms. For IgG, the “Positive by Any Assay” approach preserved excellent diagnostic performance, with sensitivity reaching 100% and near-perfect inter-assay agreement (κ = 0.88–0.98; Table 1), confirming the robustness and consistency of IgG classification across platforms. In contrast, for IgM, the same composite definition achieved maximal sensitivity (100%; Table 4) at the expense of markedly reduced specificity (69.9–77.3%; Table 4) and poor PPV (22.2–48.7%; Table 4), substantially inflating false-positive classifications. While this strategy may be suitable for epidemiological surveillance or seropositivity estimation, it is inappropriate for clinical decision-making, where inflated IgM positivity may lead to unnecessary follow-up, patient anxiety, and mismanagement. Conversely, the “Positive by All Assays” definition yielded a highly specific lower-bound estimate of IgM seropositivity, demonstrating that concordant IgM positivity across platforms is uncommon and reinforcing the absence of a perfect IgM diagnostic assay^21,53,54^. Taken together, the divergence between “Positive by Any Assay” and “Positive by All Assays” underscores that no single CMV serological assay can be considered definitive, and that composite approaches are best used to bound diagnostic uncertainty rather than to define an absolute reference standard^31–33^.

The clinical implications of these findings are significant. CMV IgG testing is reliable for determining immune status and is appropriate for antenatal screening, transplant risk stratification, and evaluation of immunocompromised individuals. However, distinguishing recent from past infection cannot be achieved using IgG kinetics alone. Portet Sulla et al. (2025) demonstrated that CMV IgG avidity testing is a more reliable marker of primary infection, whereas IgG kinetics show poor diagnostic performance^55^. Although both CLIA and ELISA platforms are widely used for CMV IgG detection, substantial inter-assay variability in IgG quantitation has been reported, with differences of up to 185-fold across systems, which may influence clinical interpretation^56^. In contrast, reliance on CMV IgM as a stand-alone marker of recent infection is limited by variability and moderate inter-assay agreement. False-positive IgM results may lead to unnecessary follow-up, increased patient anxiety, and inappropriate clinical management, particularly in pregnancy and transplantation settings^21,30^. These findings reinforce current recommendations that IgM results should be interpreted cautiously and supplemented with confirmatory testing, such as IgG avidity or molecular assays^20,21,30^.

To further assess the clinical relevance of isolated IgM reactivity, CMV IgG avidity testing was performed on 82 IgM-positive/IgG-positive samples to evaluate whether these results could represent very recent primary infection rather than nonspecific IgM reactivity. As detailed in Sect. 3.4 and summarized in Fig. 3, only two samples demonstrated low IgG avidity (< 30–40%), compatible with recent infection, and two samples showed intermediate avidity (40–60%), yielding an indeterminate result. As further mentioned in Fig. 3, strong IgG avidity predominated across IgM-positive samples for all assays, indicating that the vast majority of IgM reactivity corresponded to non-recent infection. The remaining samples lacked avidity patterns supportive of recent infection, reinforcing the limited clinical utility of IgM as a stand-alone marker. Accurate classification of primary CMV infection is particularly critical in pregnancy, as low IgG avidity reliably indicates recent infection associated with increased risk of vertical transmission, whereas high avidity early in gestation effectively excludes postconception infection and suggests a low fetal transmission risk^49^.

Within this small subset of samples with low or indeterminate avidity identified in Sect. 3.4, inter-assay classification differed across platforms. One low-avidity sample was classified as positive by Abbott and NovaLisa and as indeterminate (considered positive) by Mindray, but was reported as negative by Roche, indicating missed detection by that platform. In contrast, the second low-avidity sample and the indeterminate-avidity sample were consistently positive across all four assays. As shown in Fig. 3, Abbott CMIA identified the highest number of IgM-positive samples (*n* = 41), exceeding the counts observed with Roche, Mindray, and NovaLisa. However, avidity stratification demonstrated that the vast majority of these Abbott-positive results were associated with strong IgG avidity, indicating non-recent infection. This discordance suggests that the higher IgM positivity observed with Abbott is largely attributable to false-positive or clinically irrelevant IgM reactivity rather than true acute CMV infection. Collectively, these findings underscore that increased analytical sensitivity in IgM detection does not necessarily translate into improved clinical specificity and reinforce the need for avidity or molecular confirmation when interpreting CMV IgM results.

As a conclusion, the findings in our study highlight fundamental limitations in CMV IgM serology and confirm that no single assay provides definitive classification of recent infection, particularly in high-prevalence settings. Our results suggest prioritizing CMV IgG testing, with avidity testing, when necessary, while recognizing the limitations of IgM as a stand-alone tool due to its variability and diagnostic uncertainty. Reducing reliance on IgM in routine use may help streamline clinical workflows and reduce unnecessary follow-up.

## Strengths and limitations

A major strength of this study is its head-to-head evaluation of four widely used CMV serology platforms spanning automated CLIA/ECLIA technologies and a conventional ELISA, with assessment of both IgG and IgM and detailed inter-assay agreement analyses. The inclusion of IgG avidity testing strengthened clinical interpretation by providing an orthogonal method to clarify whether IgM reactivity plausibly reflected recent primary infection. In addition, use of a concordance-based composite silver standard reduced reliance on any single assay in a setting where a true gold standard is not available, particularly for IgM.

Limitations include incomplete testing across all platforms for some samples, which may affect pairwise comparisons and limit the direct generalizability of certain estimates. In addition, CMV IgG avidity testing was not available across all evaluated platforms and was performed exclusively using the NovaLisa CMV IgG avidity assay, which may limit cross-platform comparability of avidity results. The cohort was geographically and demographically restricted, and molecular confirmation was not systematically performed for all discordant serological profiles, which constrained definitive classification in selected cases. While molecular diagnosis of CMV, such as CMV-DNA PCR, offers high diagnostic accuracy, its high cost, labor intensity, and limited yield in large-scale screening make it impractical for routine use in antenatal or blood-donor settings. This reinforces the importance of serological testing for broader screening efforts. Future studies integrating standardized PCR testing for discordant results and applying reflex diagnostic algorithms prospectively—particularly in pregnant and immunocompromised populations—would further strengthen diagnostic interpretation and inform evidence-based testing pathways.

## Conclusion

**Fig. 4:**
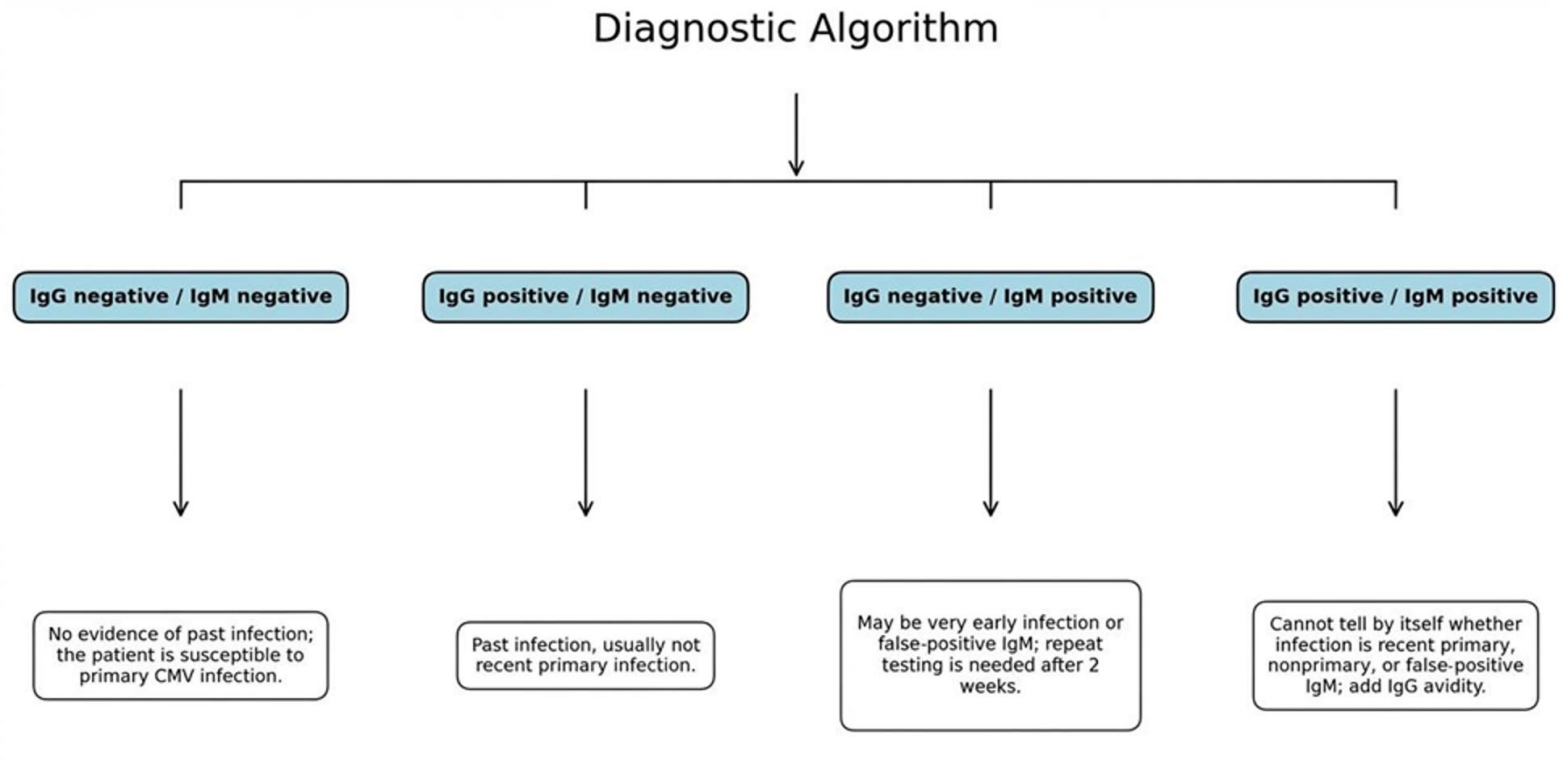
Diagnostic algorithm for CMV serological interpretation. IgG testing is used to identify past infection, while IgG avidity helps determine recent primary infection, particularly in cases with discordant results. IgM alone should not be used for diagnosis due to its limitations and should be interpreted alongside IgG and avidity to improve accuracy.

In conclusion, our study demonstrates that CMV IgG testing across both CLIA and ELISA platforms provides reliable and largely comparable diagnostic performance, supporting its use for determining immune status in clinical practice. In contrast, CMV IgM assays exhibited substantial inter-assay variability and limited concordance, underscoring their reduced reliability as stand-alone markers of recent infection. Composite analyses, including the “Positive by All Assays” definition, highlighted that concordant positivity across platforms is uncommon and that no single CMV serological assay can be considered definitive, particularly for IgM. Based on these findings, CMV IgG testing should be prioritized for routine screening, especially in pregnant and immunocompromised populations, with IgG avidity or molecular testing used to clarify suspected recent infection. Accordingly, a proposed diagnostic algorithm integrating IgG and IgG avidity testing is presented in Fig. 4. CLIA platforms offer additional operational advantages, including automation and high throughput, making them well suited for routine laboratory workflows. Diagnostic assay selection should therefore be guided by clinical context, balancing analytical reliability with efficiency in CMV serological assessment.

## Data Availability

The datasets generated and/or analyzed during the current study are available from the corresponding author on reasonable request.

## Abbreviations

CMV: Cytomegalovirus
CLIA: Chemiluminescent Immunoassay
ECLIA: Electrochemiluminescence Immunoassay
ELISA: Enzyme-Linked Immunosorbent Assay
IgG: Immunoglobulin G
IgM: Immunoglobulin M
PPV: Positive Predictive Value
NPV: Negative Predictive Value
kappa: Cohen’s Kappa Coefficient
HRP: Horseradish Peroxidase
RLUs: Relative Light Units
PCR: Polymerase Chain Reaction
ISO: International Organization for Standardization
IRB: Institutional Review Board
